# Assessment of the utility of a symptom-based algorithm for identifying febrile patients for malaria diagnostic testing in Senegal

**DOI:** 10.1186/s12936-017-1750-y

**Published:** 2017-03-01

**Authors:** Julie Thwing, Fatou Ba, Alou Diaby, Younouss Diedhiou, Assane Sylla, Guelaye Sall, Mame Birame Diouf, Alioune Badara Gueye, Seynabou Gaye, Medoune Ndiop, Moustapha Cisse, Daouda Ndiaye, Mady Ba

**Affiliations:** 1 U.S. Centers for Disease Control and Prevention and President’s Malaria Initiative, Atlanta, USA; 2 Senegal National Malaria Control Programme, Dakar, Senegal; 3 Pediatrics Service Hôpital le Dantec, Dakar, Senegal; 4 Parasitology Service Hôpital le Dantec, Dakar, Senegal; 5 United States Agency for International Development, Dakar, Senegal; 6 Université Cheikh Anta Diop, Dakar, Senegal; 7 WHO, Dakar, Senegal

**Keywords:** Malaria, Diagnosis, Treatment, Algorithm, Fever, Case management, Rapid diagnostic test

## Abstract

**Background:**

Malaria rapid diagnostic tests (RDTs) enable point-of-care testing to be nearly as sensitive and specific as reference microscopy. The Senegal National Malaria Control Programme introduced RDTs in 2007, along with a case management algorithm for uncomplicated febrile illness, in which the first step stipulates that if a febrile patient of any age has symptoms indicative of febrile illness other than malaria (e.g., cough or rash), they would not be tested for malaria, but treated for the apparent illness and receive an RDT for malaria only if they returned in 48 h without improvement.

**Methods:**

A year-long study in 16 health posts was conducted to determine the algorithm’s capacity to identify patients with *Plasmodium falciparum* infection identifiable by RDT. Health post personnel enrolled patients of all ages with fever (≥37.5 °C) or history of fever in the previous 2 days. After clinical assessment, a nurse staffing the health post determined whether a patient should receive an RDT according to the diagnostic algorithm, but performed an RDT for all enrolled patients.

**Results:**

Over 1 year, 6039 patients were enrolled and 58% (3483) were determined to require an RDT according to the algorithm. Overall, 23% (1373/6039) had a positive RDT, 34% (1130/3376) during rainy season and 9% (243/2661) during dry season. The first step of the algorithm identified only 78% of patients with a positive RDT, varying by transmission season (rainy 80%, dry 70%), malaria transmission zone (high 75%, low 95%), and age group (under 5 years 68%, 5 years and older 84%).

**Conclusions:**

In all but the lowest malaria transmission zone, use of the algorithm excludes an unacceptably large proportion of patients with malaria from receiving an RDT at their first visit, denying them timely diagnosis and treatment. While the algorithm was adopted within a context of malaria control and scarce resources, with the goal of treating patients with symptomatic malaria, Senegal has now adopted a policy of universal diagnosis of patients with fever or history of fever. In addition, in the current context of malaria elimination, the paradigm of case management needs to shift towards the identification and treatment of all patients with malaria infection.

## Background

Senegal is a malaria-endemic country in the Sahel zone on the west coast of Africa. Malaria transmission is highly variable across the country, with very low transmission in the north, increasing towards the south. Dakar, the capital city, experiences heterogeneous transmission. Transmission is highly seasonal, with the majority of cases occurring during and just after the rainy season, which generally lasts from July through October. The Senegal National Malaria Control Programme (NMCP) has rapidly scaled up malaria control interventions during the last decade. Artemisinin-based combination therapy (ACT) was introduced in 2006 [1], first at health facilities, and then at community level. Following a feasibility study [2], rapid diagnostic tests (RDTs) were introduced in late 2007, first at health facility level, and then at community level [3]. By 2009, 86% of suspected malaria cases received a diagnostic test [4], greatly decreasing the use of ACT. Microscopy is used in referral health centres and hospitals and is used almost exclusively for hospitalized patients.

**Fig. 1:**
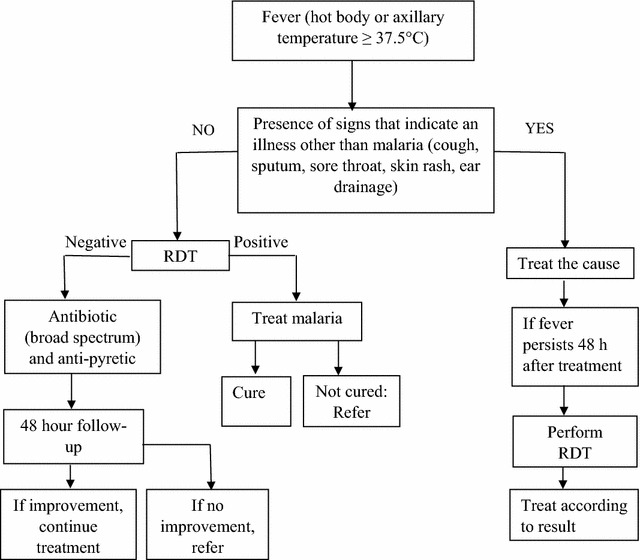
Senegal National Malaria Control Program case management algorithm for patients of all ages with uncomplicated febrile illness or history of fever in Senegal

After lengthy consultations with stakeholders, including infectious disease physicians, regional and district medical officers, other Ministry of Health officials, and financial and technical partners, a symptom-based case management algorithm was introduced, along with RDTs, which changed the definition of a suspected malaria case. In 2007, Senegal had just entered the phase of rapid scale-up of malaria control interventions, and the number of cases of fever exceeded available funding needed to test all fever cases. In view of conserving RDTs and testing those most likely to have malaria, the first step of the case management algorithm directed that not all patients with uncomplicated febrile illness or history of fever should receive an RDT. While those without an obvious fever source other than malaria were recommended to receive an RDT, those that also had symptoms or signs indicative of another source of fever (ear drainage, sore throat, sputum, cough, rash, or other fever source, according to the clinical judgment of the healthcare provider) were recommended to be treated for that indication and return for a follow-up visit 48 h later, at which point if there was no clinical improvement, an RDT was recommended. Patients with positive RDTs were to receive treatment with an ACT (Fig. 1). At community level, all cases of fever continued to be tested. The rationale behind this strategy was that even if some of these patients with fever and symptoms of another fever source did in fact have parasitaemia, it was, in effect, an asymptomatic parasitaemia, with the fever attributable to another source.

As the nation gained experience with the algorithm, concerns grew that the algorithm might miss an unacceptable number of patients with parasitaemia accompanying other symptoms, with dangerous consequences, particularly for children under 5 years old. Health workers in higher transmission zones complained that if they followed the algorithm, they missed patients with malaria. The proportion of patients with fever associated with signs suggestive of other diseases, but also suffering from malaria, was unknown.

Attempts to quantify the malaria-attributable fraction, the proportion of fevers among people with malaria infection that are actually due to malaria, have been made by a number of authors, using both cross-sectional household surveys and health facility-based patient enrolment, both to determine malaria-attributable fraction for a population through modelling and to determine age and season-based cut-offs, though this was found to vary greatly by age, season and transmission intensity [5–9]. Malaria case management has historically been based on clinical diagnosis in resource-poor settings, resulting in massive overdiagnosis and overtreatment of malaria [10–13]. Some have attempted to develop rule-based systems to diagnose malaria clinically, but the sensitivity and specificity remain poor [14–16]. There has been no other documented experience of a country adopting a case management algorithm that included clinical assessment along with fever or history of fever for recommendation of malaria testing.

This study was designed to evaluate the first step of the case management algorithm, which recommends testing for malaria if no signs or symptoms of an alternative fever source are present, as a screening test, considering the RDT as the definitive test, to determine the sensitivity, specificity, positive predictive value, and negative predictive value of limiting malaria RDT testing based on the first step of this algorithm, compared to universal RDT testing for febrile patients throughout the malaria transmission zones of Senegal and throughout the year to guide case management policy. These values were to be determined for patients under 5 years old and 5 years and above, during rainy and dry season, and in each of four malaria transmission zones.

## Methods

### Study design

**Fig. 2:**
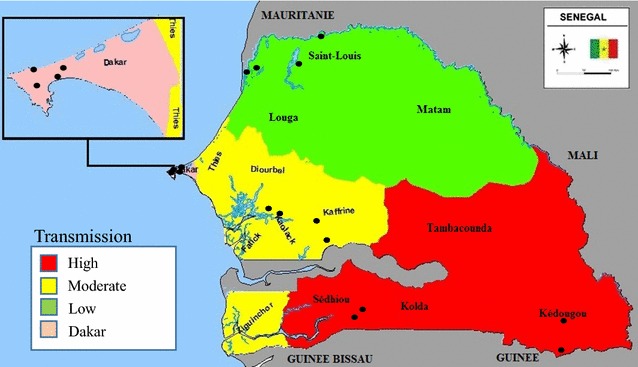
Map of study sites by transmission zone. *Filled circle* study site. Source: Senegal National Malaria Control Program

A longitudinal health facility-based study was conducted at 16 health posts in eight health districts, with two health districts in each of the four malaria transmission zones considered by the NMCP: north (low transmission, reported annual malaria incidence <5/1000 inhabitants); centre (moderate transmission, reported annual malaria incidence 5–35/1000 inhabitants); south (high transmission, reported annual incidence >35/1000 inhabitants); and, the capital city of Dakar (heterogeneous) (Fig. 2). Health posts were chosen by district health officers to have median use and to be typical of the district.

In order to have an adequate sample size to analyse results by age group (<5 and ≥5 years), season (rainy and dry), and transmission zone (high, moderate, low, heterogeneous/Dakar), with a 95% confidence interval, with a margin of 5%, at least 369 patients were required to be enrolled at each site. The sample size was set at 6144, with each of the 16 health posts expected to enrol 32 patients monthly for 12 months (384 per site).

### Enrolment

Health post staff were trained on study methods and conducted the enrolment. During a full year (April 2013–March 2014), health post staff recruited patients 4 days per week, enrolling the first patient of age at least 2 months (infants younger than 2 months are excluded from the algorithm) but younger than 5 years and the first patient 5 years or older each day of enrolment who presented with fever (axillary temperature of ≥37.5 °C) or history of fever in the previous 48 h, for their first visit to the health facility for that illness. If a febrile patient of either age group did not present on a given day of enrolment, catch-up was allowed on other days of the week. Pregnant women and patients with signs of severe disease were excluded, as they are excluded from the algorithm.

All recruited patients gave informed consent, or consent of caregiver in the case of minors. Health post staff then completed a questionnaire documenting region, district, health facility, age, gender, duration of symptoms, previous care sought, temperature, symptoms, and physical examination. Based on these, they determined whether testing with an RDT would be recommended by the algorithm, and documented their determination. Following this, all enrolled patients were tested with RDT (SD BIOLINE Malaria Ag Pf HRP-2), with results and treatment documented. During the first 9 months of the study, as an RDT quality control measure for patients enrolled on 2 of the 4 days each week, a thick blood film was also performed for quality control. All patients were treated according to the result of the RDT with an ACT for positive tests as directed by national policy, with ancillary therapy as indicated. All patients were given an appointment for follow-up in 48 h.

### Laboratory procedures

Every month, a team composed of a research assistant and a laboratory technician visited every health post, reviewed study materials, collected questionnaires and blood films, did any needed trouble shooting, and supervised and corrected blood film technique. Blood films were dried and labelled with pre-printed ID stickers on site, and were stained in 10% Giemsa for 15 min and read in Dakar by expert malaria microscopists in the national reference laboratory (University Cheikh Anta Diop Parasitology Department). Two parasitologists read the films in a blinded manner, with a third reading in case of discrepancy between the results of the two readings. In case of discrepancy, the third microscopist’s reading was used. There were discrepant readings on five slides. Asexual malaria parasites were enumerated against 200–500 white blood cells (WBC), transformed to parasites/µL using the assumption of 8000 WBC/µL; 250 fields were read before declaring any film negative.

### Data management

Questionnaires were entered into an Epi-Info (version 7.1.4, CDC, Atlanta, GA, USA) database by double entry. Every quarter, the research team met to review the data and address any data quality issues. Data were analysed using Epi Info.

The sample was restricted to patients enrolled from 1 April, 2013 to 31 March, 2014. During some months, health post personnel, trying to make up for months during which they had not enrolled the desired sample size per month, enrolled a large number of patients. Up to double the month’s quota of 32 were accepted. For any month during which a site enrolled more than 64 patients, enrollees were randomly selected from the highest enrolment days for removal. This exercise was performed for three health posts: two during July and one during August.

### Analysis

Rainy season was defined as July–December and dry season was defined as January–June. Fever was defined as an axillary temperature of ≥37.5 °C, and history of fever was defined as a complaint of a hot body in the previous 48 h. In order to determine if health providers had implemented the algorithm and correctly assigned patients to the groups to be tested, two senior members of the research team reviewed the symptoms recorded for each patient and determined whether the algorithm recommended testing. Concordance between health post workers and expert review was assessed. Sensitivity, specificity, positive predictive value, and negative predictive value of the algorithm as a screening test were calculated, compared to RDT. This calculation was done across age groups, transmission zones and rainy/dry season to determine for which age groups, in which transmission zones, and for which seasons the algorithm demonstrated sufficient sensitivity to diagnose an adequate proportion of febrile patients with parasitaemia.

### Ethical aspects

The protocol was reviewed and approved by the Senegal National Ethical Committee (*Comité National d’Ethique pour la Recherche Santé #0109*) and by the Centers for Disease Control and Prevention Human Subjects Board, where it received a non-research determination. Participation was strictly voluntary. Patients who refused consent received the same evaluation and treatment as they would have if they had participated, other than an RDT for patients that would not have met criteria for RDT according to the algorithm.

## Results

### Enrolment and demographics

From April 2013 to March 2014, 6039 patients were enrolled. Of these, 46.5% (95% CI 45.2–47.7) were children under five, reflective of the instructions given, and 48.4% were male (95% CI 47.2–49.7). Of the 5988 for whom data were recorded, 32.2% (95% CI 31.0–33.4) sought initial care elsewhere prior to this consultation. Of 6035 for whom temperature was recorded, 83.1% (95% CI 82.1–84.0) were febrile at consultation, with a median duration of symptoms of 2.4 days at the time of consultation. The breakdown by quarter and malaria risk zone is found in Table 1. While approximately equal numbers of patients were enrolled in each risk zone, 44.1% of patients were enrolled during the dry season (January–June), compared to 55.9% during the rainy season (July–December).

**Table 1:** Distribution of patients enrolled by trimester and malaria transmission risk zone

	Jan–Mar	Apr–Jun	Jul–Aug	Oct–Dec	Total
North (low)	248	371	443	371	1433 (24%)
Centre (moderate)	350	394	418	376	1538 (25%)
South (high)	261	366	533	334	1494 (25%)
Dakar (heterogeneous)	320	353	522	378	1573 (26%)
Total	1179 (20%)	1484 (25%)	1916 (32%)	1459 (24%)	6038

### Need for testing as determined by algorithm

Overall, 57.7% (95% CI 56.4–58.9) (3483/6039) were determined eligible to receive an RDT according to the algorithm (Fig. 1), which varied by age group, season and malaria transmission risk zone (Table 2). Of children under 5 years, 44.4% (95% CI 42.6–46.3) were determined eligible to be tested, compared to 69.2% (95% CI 67.6–70.8) of patients 5 years or older. The percent of patients eligible to be tested by malaria risk zone ranged from 50.8% (heterogeneous) to 64.5% (high). With the exception of the high transmission risk zone, in which 54.2% (95% CI 44.8–52.4) were to be tested in dry season compared to 72.0% (95% CI 68.8–74.9) in rainy season, the difference from rainy to dry season was minimal. Concordance between the health post workers and study team regarding patients requiring testing according to the algorithm was 90.0% (95% CI 89.2–90.7).

**Table 2:** Percent of febrile patients qualifying for rapid diagnostic testing according to the algorithm, by age group, transmission season, and malaria transmission risk zone

Season	Under 5 years			At least 5 years			All ages		
Stratum (risk zone)	Dry	Rainy	All	Dry	Rainy	All	Dry	Rainy	All
	Percent (LCL–UCL)	Percent (LCL–UCL)	Percent (LCL–UCL)	Percent (LCL–UCL)	Percent (LCL–UCL)	Percent (LCL–UCL)	Percent (LCL–UCL)	Percent (LCL–UCL)	Percent (LCL–UCL)
North (low)	46.0 (40.2–51.8) 136/296	44.8 (39.8–50.0) 169/377	45.3 (41.5–49.2) 305/673	60.1 (54.5–65.4) 194/323	63.2 (58.4–67.7) 276/437	61.8 (58.3–65.3) 470/760	53.3 (49.3–57.3) 330/619	54.7 (51.2–58.1) 445/814	54.1 (51.5–56.7) 775/1433
Centre (moderate)	46.2 (41.0–51.4) 170/368	47.6 (42.6–52.6) 189/397	46.9 (43.4–50.5) 359/765	75.5 (70.8–79.7) 284/376	76.1 (71.5–80.1) 302/398	75.7 (72.5–78.7) 586/774	61.0 (57.4–64.5) 454/744	61.8 (58.4–65.2) 491/795	61.4 (58.9–63.8) 945/1539
South (high)	43.8 (38.1–49.7) 128/292	59.6 (54.6–64.4) 236/396	52.9 (49.1–56.7) 364/688	63.3 (57.9–68.4) 212/335	82.4 (78.6–85.7) 388/471	74.4 (71.3–77.4) 600/806	54.2 (50.2–58.2) 340/627	72.0 (68.8–74.9) 624/867	64.5 (62.0–66.9) 964/1494
Dakar (heterogeneous)	33.6 (28.0–39.5) 92/274	31.0 (26.6–35.8) 126/406	32.1 (28.6–35.7) 218/680	58.9 (53.9–63.7) 235/399	70.0 (65.8–74.0) 346/494	65.1 (61.8–68.2) 581/893	48.6 (44.8–52.4) 327/763	52.4 (49.1–55.8) 472/900	50.8 (48.3–53.3) 799/1573
All	42.8 (40.0–45.6) 526/1230	45.7 (43.2–48.2) 720/1576	44.4 (42.6–46.3) 1246/2806	64.6 (62.0–67.0) 925/1433	72.9 (70.8–75.0) 1312/1800	69.2 (67.6–70.8) 2237/3233	54.5 (52.6–56.4) 1451/2663	60.2 (58.5–61.9) 2032/3376	57.7 (56.4–58.9) 3483/6039

*LCL* lower confidence limit, *UCL* upper confidence limit

### Rapid diagnostic test positivity

Regardless of whether they were eligible for an RDT or not according to the algorithm, all enrolled patients were tested for malaria with an RDT; results were available for 6038/6039. Overall, 22.7% (95% CI 21.7–23.8) were positive for malaria. RDT positivity rate was 11.7% (95% CI 10.5–13.0) (298/2554) for those for whom an RDT was not recommended by the first step of the algorithm, compared to 30.9% (95% CI 29.4–32.4) (1075/3483) for those for whom an RDT was recommended by the algorithm. Children under 5 years had an RDT positivity rate of 16.5% (95% CI 15.1–17.9) compared to 28.2% (95% CI 26.7–29.8) for patients 5 years or older. The positivity rate noted by risk zone confirmed the highly stratified epidemiology. In the low risk northern zone, the RDT positivity rate was only 1.3% (95% CI 0.8–2.1), varying from 0.5% (95% CI 0.1–1.5) in the dry season to 2.0% (95% CI 1.2–3.2.) in the rainy season. In the high-risk southern zone, the RDT positivity rate was 56.5% (95% CI 53.9–59.0), varying from 27.1% (95% CI 23.7–30.9) in the dry season to 77.6% (95% CI 74.7–80.3) in the rainy season. The centre (moderate) and Dakar (heterogeneous) were intermediate in RDT positivity, with a five-fold difference from dry to rainy season (Table 3).

**Table 3:** Percent of febrile patients positive for malaria by RDT, by age group, transmission season, and malaria transmission risk zone

Season	Under 5 years			At least 5 years			ALL ages		
Stratum (risk zone)	Dry	Rainy	All	Dry	Rainy	All	Dry	Rainy	All
	Percent (LCL–UCL)	Percent (LCL–UCL)	Percent (LCL–UCL)	Percent (LCL–UCL)	Percent (LCL–UCL)	Percent (LCL–UCL)	Percent (LCL–UCL)	Percent (LCL–UCL)	Percent (LCL–UCL)
North (low)	0.3 (0.0–1.9) 1/296	1.3 (0.5–3.3) 5/377	0.9 (0.4–2.0) 6/673	0.6 (0.1–2.5) 2/323	2.5 (1.3–4.6) 11/437	1.7 (1.0–3.0) 13/760	0.5 (0.1–1.5) 3/619	2.0 (1.2–3.2) 16/814	1.3 (0.8–2.1) 19/1433
Centre (moderate)	5.2 (3.2–8.1) 19/368	21.9 (18.0–26.4) 87/397	13.9 (11.5–16.6) 106/765	6.9 (4.7–10.1) 26/376	39.0 (34.3–44.1) 155/242	23.4 (20.5–26.6) 181/774	6.1 (4.5–8.1) 45/744	30.5 (27.3–33.8) 242/795	18.7 (16.8–20.7) 287/1539
South (high)	18.8 (14.5–23.8) 55/292	68.4 (63.6–72.9) 271/396	47.4 (43.6–51.2) 326/688	34.4 (29.4–39.8) 115/334	85.4 (81.8–88.4) 402/471	64.2 (60.8–67.5) 517/805	27.2 (23.7–30.9) 170/626	77.6 (74.7–80.3) 673/867	56.5 (53.9–59.0) 843/1493
Dakar (heterogeneous)	2.9 (1.3–5.7) 8/273	3.9 (2.4–6.5) 16/406	3.5 (2.3–5.3) 24/679	4.3 (2.6–6.9) 17/399	37.0 (32.8–41.5) 183/494	22.4 (19.7–25.3) 200/893	3.7 (2.5–5.5) 25/672	22.1 (19.5–25.0) 199/900	14.3 (12.6–16.1) 224/1572
All	6.8 (5.4–8.3) 83/1229	24.1 (22.0–26.3) 379/1576	16.5 (15.1–17.9) 462/2805	11.2 (9.6–13.0) 160/1432	41.8 (39.5–44.1) 751/1800	28.2 (26.7–29.8) 911/3232	9.1 (8.1–10.3) 243/2661	33.5 (31.9–35.1) 1130/3376	22.7 (21.7–23.8) 1373/6037

*LCL* lower confidence limit, *UCL* upper confidence limit

### Microscopy for RDT quality assurance

During the first 9 months of the study (April–December 2013), blood films were collected for 49.3% of patients (2396/4856), and 23.3% (21.7–25.1) of blood films were positive, compared to 25.5% (95% CI 24.3–26.7) of RDTs from this period. All positive samples were identified as *Plasmodium falciparum*; no other malaria parasite species were detected by microscopy. Sensitivity and specificity of RDTs, compared to blood films (of the 2396 RDTs performed during the first 9 months), were both 99.1%, demonstrating excellent reliability in the hands of well-trained health post staff.

### Characteristics of the diagnostic algorithm

Overall, sensitivity of the first step of the diagnostic algorithm (based on facility health workers’ assessments) compared to RDT was 78.3% (95% CI 76.0–80.4), specificity was 48.4% (95% CI 46.9–49.8), negative predictive value was 88.3% (95% CI 87.2–89.4), and positive predictive value was 30.9% (95% CI 30.0–31.7). Calculation of the diagnostic values for both age groups, rainy and dry season, and every transmission risk zone separately demonstrated large differences in diagnostic values depending on these categories (Table 4). The sensitivity of the algorithm followed transmission intensity, with the greatest sensitivity (but wide confidence intervals, due to low numbers of malaria cases) in areas of lowest transmission (north), progressively decreasing with increasing transmission, and the lowest sensitivity (and narrower confidence intervals) in the south. Sensitivity in the north, calculated for the entire year, was 83.3% (95% CI 36.9–99.6) among children under 5 years and 100.0% (95% CI 75.3–100.0) among patients 5 years and older. By comparison, in the south, sensitivity calculated for the entire year was 64.7% (95% CI 59.3–70.0) among children under 5 years, and 81.0% (95% CI 77.4–84.3) among patients 5 years and older. The moderate transmission centre fell between these, with sensitivity calculated for year of 75.5% (95% CI 66.2–83.3) among children under 5 years and 85.1% (95% CI 76.6–85.9) among patients 5 years and older. In every season and risk zone, sensitivity among children under 5 years was less than that for patients 5 years and older, with sensitivity calculated all year and nationwide of 67.5% (95% CI 63.1–71.8) among children under 5 years and 83.8% (95% CI 81.2–86.1) among patients 5 years and older. No clear seasonal trend regarding sensitivity emerged. Outside the north, sensitivity did not surpass 90% in any age group, season or risk zone. While specificity was low throughout, as the first step of the algorithm is used as a screening test, a certain level of false positivity is desirable.

**Table 4:** Diagnostic values of case management algorithm, by age group, transmission season, and malaria transmission risk zone

Risk zone	Age group (years)	Season	TP	FP	FN	TN	SENS % (95% CI)	SPEC % (95% CI)	NPV % (95% CI)	PPV % (95% CI)
North (low)	<5	Dry	1	135	0	160	100.0 (2.5–100.0)	54.2 (48.4–60.0)	100.0 (^a^)	0.7 (0.7–0.8)
		Rainy	4	165	1	207	80.0 (28.4–99.5)	55.6 (50.4–60.8)	99.5 (97.3–99.9)	2.4 (1.5–3.7)
		All year	5	300	1	367	83.3 (36.9–99.6)	55.0 (51.2–58.8)	99.7 (98.4–100.00)	1.6 (1.1–2.4)
	≥5	Dry	2	192	0	129	100.0 (15.8–100.0)	40.2 (34.8–45.8)	100.0 (^a^)	1.0 (0.9–1.1)
		Rainy	11	265	0	161	100.0 (71.5–100.0)	37.8 (33.2–42.6)	100.0 (^a^)	4.0 (3.7–4.3)
		All year	13	457	0	290	100.0 (75.3–100.0)	38.8 (35.3–42.4)	100.0 (^a^)	2.8 (2.6–2.9)
Centre (mod)	<5	Dry	14	156	5	193	73.7 (48.8–90.9)	55.3 (49.9–60.6)	97.5 (94.5–98.8)	8.2 (6.3–10.7)
		Rainy	66	123	21	187	75.9 (65.5–84.4)	60.3 (54.6–65.8)	89.9 (85.9–92.9)	34.9 (30.9–39.2)
		All year	80	279	26	380	75.5 (66.2–83.3)	57.7 (53.8–61.5)	93.6 (91.2–95.4)	22.3 (20.0–24.8)
	≥5	Dry	22	262	4	88	84.6 (65.1–95.6)	25.1 (20.7–30.0)	95.7 (89.8–98.2)	7.7 (6.6–9.1)
		Rainy	132	170	23	72	85.2 (78.6–90.4)	29.8 (24.1–35.9)	75.8 (67.2–82.7)	43.7 (41.1–46.3)
		All year	154	432	27	160	85.1 (79.0–89.9)	27.0 (23.5–30.8)	85.6 (80.3–89.6)	26.3 (24.8–27.8)
South (high)	<5	Dry	28	100	27	137	50.9 (37.1–74.7)	57.8 (51.2–64.2)	83.5 (79.2–87.2)	21.9 (17.2–27.4)
		Rainy	183	53	88	72	67.5 (61.6–73.1)	57.6 (48.4–66.4)	45.0 (39.4–50.7)	77.5 (73.5–81.2)
		All year	211	153	115	209	64.7 (59.3–70.0)	57.7 (52.5–62.9)	64.5 (60.5–68.3)	58.0 (54.4–61.4)
	≥5	Dry	80	132	35	87	69.6 (60.3–77.8)	39.7 (33.2–46.5)	71.3 (64.3–77.4)	37.7 (34.0–41.6)
		Rainy	339	49	63	20	84.3 (80.4–87.7)	29.0 (18.7–41.2)	24.1 (17.1–32.9)	87.4 (85.5–89.0)
		All year	419	181	98	107	81.0 (77.4–84.3)	37.2 (31.6–43.0)	52.2 (46.4–58.0)	69.8 (67.7–71.9)
Dakar (mixed)	<5	Dry	7	85	1	180	87.5 (47.4–99.7)	67.9 (61.9–73.5)	99.4 (96.6–99.9)	7.6 (5.7–10.1)
		Rainy	9	117	7	273	56.3 (29.9–80.3)	70.0 (65.2–80.3)	97.5 (95.7–98.6)	7.1 (4.6–10.8)
		All year	16	202	8	453	66.7 (44.7–84.4)	69.2 (65.5–72.7)	98.3 (97.0–99.0)	7.3 (5.5–9.7)
	≥5	Dry	15	220	2	162	88.2 (63.6–98.5)	42.4 (37.4–47.5)	98.8 (95.6–99.7)	6.4 (5.3–7.6)
		Rainy	162	184	21	127	88.5 (83.0–92.8)	40.8 (35.3–46.5)	85.8 (79.8–90.2)	46.8 (44.2–49.5)
		All year	177	404	23	289	88.5 (83.3–92.6)	41.7 (38.0–45.4)	92.6 (89.4–94.9)	30.5 (28.8–32.2)

TP, true positive: algorithm recommends testing, and RDT is positive; FP, false positive: algorithm recommends testing, and RDT is negative; FN, false negative: algorithm does not recommend testing, and RDT and positive; TN, true negative: algorithm does not recommend testing, and RDT is negative

*SENS* sensitivity, *SPEC* specificity, *NPV* negative predictive value, *PPV* positive predictive value

^a^Unable to calculate

Both positive and negative predictive values were highly associated with season and malaria transmission risk zone. Negative predictive value was high during the dry season and in lower transmission zones, and was consistently higher among children under 5 than among patients 5 years and older. With the exception of the high transmission south, it was consistently greater than 95% during dry season and above 75% in rainy season. In the south during the rainy season, negative predictive value plunged to 24.8% (95% CI 17.1–32.9) among patients 5 years and older, and 45.0% (95% CI 39.4–50.7) among children under 5 years. Given that malaria incidence is generally low in Senegal, positive predictive value was relatively low, save in the south during rainy season, where it was 77.5% (95% CI 73.5–81.2) among children under 5 years and 87.4% (85.5–89.0) among patients 5 years and older.

The sensitivity of the study team’s determination of RDT eligibility according to the algorithm compared to RDT was determined to be 82.8% (95% CI 80.7–84.8), compared to 78.3% (95% CI 76.0–80.4) for the determination by the health post workers. Specificity was 39.4% (95% CI 38.0–40.8), compared to 48.4% (95% CI 46.9–49.8) for the health post workers. Other diagnostic values were within 5% points.

Considering only patients for whom a blood film was positive (n = 621), 22% (137) had been judged not to be eligible to receive an RDT. Patients for whom a blood film was positive but who were judged not eligible to receive an RDT had a mean parasite density of 23,075 parasites/µL (range 480–288,000), while patients with positive smear who had been judged eligible to receive an RDT had a mean parasite density of 22,639 parasites/µL (range 400–408,000). For all positive smears, mean and median parasite density were similar for children under 5 years (mean 22,176; median 5000), children 5–9 years (mean 26,324; median 5078), and patients 10 years and older (mean 21,636; median 6045).

### Follow-up

All patients were given a 48-h follow-up appointment, which 70.0% kept. Only eight (0.2%) were judged to have had a non-favourable evolution, two of whom had had a positive RDT and had been treated with ACT. Of the five patients that had a negative RDT but positive blood film on the initial visit, parasite density was available for four and ranged from 667 to 10,160 parasites/µL at the initial visit. Four of the five attended the 48-h follow-up visit, and were all judged to have had a favourable clinical evolution.

## Discussion

Senegal was one of the first countries in sub-Saharan Africa to recommend laboratory-confirmed malaria diagnosis at all health posts nationwide and was forced to do so in the context of resource constraints. The case management algorithm, as it was designed, represented the consensus of malaria scientists and programme managers in Senegal as the best way to use limited resources to diagnose the greatest number of malaria sufferers. It was implemented with the intention to evaluate and modify it as the situation evolved.

This study was the first to measure year-round malaria test positivity rate among patients with uncomplicated febrile illness throughout Senegal, and confirms what has been seen in the routine health information system and cross-sectional surveys regarding the highly seasonal and graduated nature of malaria transmission in Senegal. The RDT positivity rate was very low during dry season and in the north, and quite high in the south in rainy season. While the proportion of patients eligible to be tested according to the algorithm (patients without another fever source) varied by transmission risk zone, the variation was within 15% points from north to south, and the variation between dry and rainy transmission seasons was only 5% points, with roughly 60% of febrile patients recommended for testing according to the algorithm.

Surprisingly, children under 5 years old were substantially less likely than patients 5 years and older to be determined eligible for a test, though young children have frequent fevers due to many upper respiratory and other infections, which diminish with age, while adults have fever more rarely, and those fevers may be more likely to be considered malarial.

While this study was not an attempt to estimate malaria-attributable fraction, the diagnostic algorithm may be considered a method to help healthcare providers identify patients among whom fever is likely to be attributable to malaria. The RDT positivity rate was indeed higher, by almost three-fold, among those recommended for RDT testing than among those not recommended for RDT testing by the first step of the algorithm. Studies that examined the effect of seasonality found a higher malaria-attributable fraction during the rainy season [7, 9] and studies that looked at the effect of transmission intensity found that a higher proportion of fevers in higher transmission zones were due to malaria [5, 6, 8], as was found in Senegal. These assumptions were generally confirmed by the proportion of positive tests by season and transmission zone. However, the findings both that febrile children under 5 years were judged by healthcare providers to be more likely to have a fever source other than malaria and that children under 5 years actually did have a lower test positivity rate stand in contrast to other studies, regardless of transmission zone. In neighbouring Mali and Burkina Faso, as well as in Kenya and Mozambique, younger children had a higher malaria-attributable fraction for fever and higher test positivity rates than older children and adults [5, 7, 9, 17]. Moreover, febrile children under 5 years had a lower test positivity rate than febrile patients 5 years and older during both rainy and dry seasons in every transmission zone in this study.

While sensitivity and specificity for laboratory tests are theoretically intrinsic and unchanging values, for a case management algorithm there is variation; this algorithm is a way of assigning testing to those with higher pre-test probability. The sensitivity of the algorithm for identifying patients with malaria among patients under 5 years was lower than that for patients 5 years or older, despite the lower test positivity rate among children under 5 years, and was lowest in the highest transmission risk zone. While the diagnostic algorithm permits testing and diagnosis of almost 80% of febrile patients with malaria parasites nationwide over the course of the year, this proportion is unacceptably low for use as a screening test. In addition, in high transmission zones during the dry season, just over one half of children under 5 years with parasites would be detected by the diagnostic algorithm, validating the complaints of the health providers that if they follow the algorithm, they miss too many cases of malaria. Low specificity, on the other hand, would not be as problematic for a screening test. Negative predictive value is generally greater than 85%, consistent with relatively low burden, except for in the high transmission zone during the rainy season, where it falls to only 45 and 24% among children under 5 years and among patients 5 years and older, respectively; while otherwise low, positive predictive value is greater than 75% among all ages in the high transmission zone during rainy season.

Some of the patients ineligible for a test according to the diagnostic algorithm that actually had malaria parasites may in fact have had symptoms due to another cause, with simultaneous asymptomatic malaria parasitaemia. When the algorithm was adopted, it was assumed that these patients likely had low density parasitaemia not requiring treatment. However, among patients for whom a blood film was obtained, and among whom that film was positive, patients for whom an RDT was recommended and patients for whom an RDT was not recommended had very similar mean parasite densities. As Senegal moves toward the goal of pre-elimination, every opportunity to diagnose and treat patients with parasites becomes increasingly important. Senegal is adopting strategies of reactive active case detection, even testing asymptomatic household contacts in elimination zones [18]. Theoretically, as exposure to parasites decreases and immunity wanes, the likelihood of truly asymptomatic parasitaemia decreases, although this decrease has not been found to be as profound as expected; evidence suggests that 60% of infections, even at low transmission, are asymptomatic [19].

This study had several limitations. Due to budgetary constraints, health post nurses were trained to carry out the study procedures instead of hiring dedicated study staff. Hence, a simplified enrolment scheme was used, and enrolment was dependent on the care providers’ comprehension of procedures as well as work schedule, though it enabled understanding of how the care providers were evaluating patients. Providers struggled to enrol the planned numbers of patients during the dry season, and among children under 5 years, and were not required to report total numbers of patients or total numbers of febrile patients seen on days of enrolment. Results were presented by age group, season and transmission stratum separately to minimize bias due to this. The limitation to 16 health posts made it impossible to get a nationally representative sample, however sites from across all four of the malaria transmission risk zones were included. While relying on a care provider to assess the need for an RDT according to the algorithm may have been a weakness, the classification by study personnel, including a member of the NMCP, had good concordance with that of the provider. Concern for another potential weakness, that of using RDT for gold standard diagnosis, was mitigated by the very high sensitivity and specificity of RDT compared to reference laboratory microscopy for the half of the samples for which a blood slide was obtained.

## Conclusions

Despite the limitations, this study provided unequivocal evidence that the first step of the case management algorithm caused providers who followed it strictly to miss an unacceptable proportion of malaria infections. Based on the poor sensitivity of the diagnostic algorithm, the Senegal NMCP is phasing out the use of the first step of the algorithm in favour of universal testing of febrile patients, while rolling out the policy over several years to assure that adequate RDT supplies could be ordered to support the new policy. Starting in 2015, all febrile children under 5 years nationwide and year-round were to be tested with an RDT, while universal testing for febrile patients 5 years or older was limited to the rainy season. The routine malaria information system in Senegal demonstrated that the number of children under 5 years tested for malaria increased from 136,847 in 2014 to 364,771 in 2015 [20, 21]. Starting in 2017, febrile patients of all ages will be tested for malaria throughout the year.
